# On temporal scale-free non-periodic stimulation and its mechanisms as an infinite improbability drive of the brain’s functional connectogram

**DOI:** 10.3389/fninf.2023.1173597

**Published:** 2023-05-24

**Authors:** Vinícius Rosa Cota, Sérgio Augusto Vieira Cançado, Márcio Flávio Dutra Moraes

**Affiliations:** ^1^Rehab Technologies - INAIL Lab, Istituto Italiano di Tecnologia, Genoa, Italy; ^2^Laboratory of Neuroengineering and Neuroscience, Department of Electrical Engineering, Federal University of São João del-Rei, São João del Rei, Brazil; ^3^Núcleo Avançado de Tratamento das Epilepsias (NATE), Felício Rocho Hospital, Fundação Felice Rosso, Belo Horizonte, Brazil; ^4^Department of Physiology and Biophysics, Núcleo de Neurociências, Federal University of Minas Gerais, Belo Horizonte, Brazil

**Keywords:** electrical stimulation, neuromodulation, coincidence-detection, temporal pattern, power-law, phase coupling, synchronization, neural circuits

## Abstract

Rationalized development of electrical stimulation (ES) therapy is of paramount importance. Not only it will foster new techniques and technologies with increased levels of safety, efficacy, and efficiency, but it will also facilitate the translation from basic research to clinical practice. For such endeavor, design of new technologies must dialogue with state-of-the-art neuroscientific knowledge. By its turn, neuroscience is transitioning—a movement started a couple of decades earlier—into adopting a new conceptual framework for brain architecture, in which time and thus temporal patterns plays a central role in the neuronal representation of sampled data from the world. This article discusses how neuroscience has evolved to understand the importance of brain rhythms in the overall functional architecture of the nervous system and, consequently, that neuromodulation research should embrace this new conceptual framework. Based on such support, we revisit the literature on standard (fixed-frequency pulsatile stimuli) and mostly non-standard patterns of ES to put forward our own rationale on how temporally complex stimulation schemes may impact neuromodulation strategies. We then proceed to present a low frequency, on average (thus low energy), scale-free temporally randomized ES pattern for the treatment of experimental epilepsy, devised by our group and termed NPS (Non-periodic Stimulation). The approach has been shown to have robust anticonvulsant effects in different animal models of acute and chronic seizures (displaying dysfunctional hyperexcitable tissue), while also preserving neural function. In our understanding, accumulated mechanistic evidence suggests such a beneficial mechanism of action may be due to the natural-like characteristic of a scale-free temporal pattern that may robustly compete with aberrant epileptiform activity for the recruitment of neural circuits. Delivering temporally patterned or random stimuli within specific phases of the underlying oscillations (i.e., those involved in the communication within and across brain regions) could both potentiate and disrupt the formation of neuronal assemblies with random probability. The usage of infinite improbability drive here is obviously a reference to the “The Hitchhiker’s Guide to the Galaxy” comedy science fiction classic, written by Douglas Adams. The parallel is that dynamically driving brain functional connectogram, through neuromodulation, in a manner that would not favor any specific neuronal assembly and/or circuit, could re-stabilize a system that is transitioning to fall under the control of a single attractor. We conclude by discussing future avenues of investigation and their potentially disruptive impact on neurotechnology, with a particular interest in NPS implications in neural plasticity, motor rehabilitation, and its potential for clinical translation.

## 1. Introduction

The powerful intuition that electricity applied to the body may bear therapeutic effects in many different ailments is much older than the knowledge of moving electrical charges itself, the potentials created by their separation, and all the related electromagnetic phenomena. Roman physicians prescribed Torpedo Ray Fishes to treat chronic pain and, before them, ancient Greeks used electrically charged amber collars with therapeutic intentions toward all natural and even unnatural afflictions (Gildenberg, 2006). Therefore, as it happens to therapeutic approaches with ancient roots, there was little to no theoretical basis to explain, or even to hypothesize on possible reasons, as to why it actually worked.

Following original insights of early investigators of bioelectrical phenomenal, such as Luigi Galvani and Alessandro Volta, and the development of rudimentary electrical devices (e.g., Leyden Jar and Van de Graaf’s generator), an era of wide application of electricity as therapy quickly ensued. At this point, controlled demonstrations of the effects of electricity on biological tissue, both dead or alive (as in Giovanni Aldini’s and Guillaume Duchenne’s demonstrations), evoked at the will of the experimenter/therapist and observable to the naked eye, left no doubt that it could be used to elicit involuntary and targeted responses in an organism. Here, the early reasoning would state that if in fact, the human body could respond to electricity through some sort of electricity “receptor”, there should also be an endogenous electricity generator to account for its existence, a process later termed bioelectrogenesis.

As often occurs after a scientific breakthrough, the rapid changes in paradigm not only spurred charlatanism—particularly during the 19th century—but also inspired notable sound technological and scientific progress. This story was extensively shared with the fields of neurology and neurosurgery, even before they were organized as areas of specific medical activity. Based on animal models and experimentation guided by cases, or even without so much scientific rigor, several physicians acted as electrotherapists. Among them, Jean-Martin Charcot laid the foundations of what many consider modern clinical neurology at the Salpêtrière Hospital, while Guillaume Duchenne consolidated electrotherapy as a treatment for diseases of the nervous system (Hagner, 2012). In fact, studies with electrostimulation of the brain were essential for supporting the localizationist theory, in which each brain function can be bi-univocally correlated to a brain area. In addition, the sometimes-conflicting interactions between physiologists and physicians led to the introduction of ES as a basic neurophysiology technique in clinical practice for questioning and confirming preclinical models. An obvious example of the great impact of this interdisciplinarity is Wilder Penfield’s studies on adapting and applying electrical stimulation techniques to patients submitted to neurosurgical procedures, which lead to the human sensory-motor cortex topographical mapping [i.e., the sensory and motor homunculus (Penfield and Boldrey, 1937; Penfield and Jasper, 1954)].

The therapeutic application of electrical (and magnetic) fields to the brain is today a very well-established medical and scientific practice, known as neuromodulation or neurostimulation (Bao et al., 2020; Krauss et al., 2021; Foutz and Wong, 2022). In fact, the field bears testimony to its historical roots, displaying sometimes the content of trial-and-error practice, but also solid principles and protocols based on an ever-evolving conceptual framework in neurosciences (Hopkins et al., 1992; von der Malsburgl, 1994; Bennett, 1999; Varela et al., 2001). This conceptual framework is in the kernel for our understanding of the very nature of information processing within the nervous system, how the brain works, and the physiopathology of its dysfunctions. As reminded by one of the greatest geniuses of humanity’s past, Leonardo da Vinci, “He who loves practice without theory is like the sailor who boards ship without a rudder and compass and never knows where he may cast.”.

In Section “2. The ever-evolving neuroscientific framework” of this review, we revisit the evolution of such a conceptual framework which establishes, in our understanding, the guiding principles that pave the way for ever-more efficacious neurostimulation methods based on evidence and sitting on a solid theoretical foundation. Along with the advised practice of full disclosure, the authors have a particular interest in the aspect of temporal patterning, a considerably more recent strategy used in stimulation protocols. Its use derives from the knowledge scaffold supporting modern neurosciences, and how it relates to a more recently proposed coincidence-detection cerebral architecture (Hopkins et al., 1992; Abeles et al., 2004) rather than the more conventional integrate and fire model of neuronal processing (not to confound with IaF computational model) (McCulloch and Pitts, 1943; Rosenblatt, 1958). Therefore, it is not surprising that the review ends with the authors putting forward their own contributions to the field stemming from the proposition of a temporally complex pattern of ES to suppress seizures and treat epilepsy, termed Non-periodic Stimulation (or NPS); as well as using ES for probing neuronal circuitry for diagnostic purposes. NPS is a non-standard form of ES in which the interpulse intervals (IPI) are randomized in a unitary exponent power-law fashion with a low average frequency (4 pulses per second). It has been shown to have robust anticonvulsant properties in animal models of acute seizure and also in animal models of epilepsy, in which permanent changes to neural tissue is induced so that seizures spontaneously occur in a chronic fashion. Mechanistic studies suggests NPS has a synchronization buffer effect (i.e., it maintains homeostatic levels, protecting against hypersynchronization), while also preserving neural function (de Oliveira et al., 2019). It is important to highlight here that research data from experiments designed to explain how such neuromodulation protocols worked only found a solid theoretical background under the coincidence detection perspective. These findings will be reviewed and discussed in Sections “3. Novel conceptions of the time dimension in the design of neuromodulation approaches” and “4. NPS meets the coincidence-detection framework: driving the infinite improbability of the brain’s functional connectogram” of this text.

The lessons we learned from applying such temporally complex stimulation protocol for treating epilepsy in experimental animal models have fostered a diversity of investigational and developmental venues, some of which are purely physiological and have no association with brain dysfunctions. These include memory engrams; a further in-depth investigation of the mechanisms of NPS itself under the light of a coincidence detection conceptual framework; the implications of the effects of the therapy in the current neuroscientific understanding of the brain functioning and disorders; novel therapeutic applications toward distinct neurological disorders including pathological anxiety, Parkinson’s, and motor deficits following stroke, and; translational research in human patients. The group is currently carrying out all these endeavors and such perspectives will be presented as a final discussion in Section “5. Discussion” of this article.

## 2. The ever-evolving neuroscientific framework

### 2.1. Beginning with the integrate and fire architecture

Even rudimentary electricity devices such as Leyden Jar and Van de Graaf’s Generator, although quite popular as a handful of parlor tricks and quackery, are methods and tools that had noteworthy importance for evolving a conceptual framework to explain functions and diseases of the brain. To highlight just a few breakthroughs: the understanding that isolated nerves (Jan Swammerdam; 1637-80) and muscles (Albrecht von Haller; 1734) were electrically “irritable”—see Pearce’s Historical Notes (Pearce, 1997), that the spinal cord was an important pathway for activating “body-motion”, and the important role cortical electrostimulation played in establishing the idea—championed by Santiago Ramon y Cajal—of brain architecture based on functional neuroanatomy (Fritsch and Hitzig, 1870; Ferrier, 1890; Jackson, 1898; Brodmann, 1909; see for historical review Finger, 1994; Levine, 2007; Molnár and Brown, 2010). All these contributions lead to the seminal work of Sir Charles Sherrington (e.g., “The integrative action of the nervous system”, with notable contributions of outstanding scientists such as Marshall Hall, Edgar Adrian, and Yngve Zotterman) which helped shape a hierarchical functional organization of the brain as layers upon layers of sensory-motor circuits, with respective modulatory inputs from several multimodal brain areas, coordinating an increasingly complex network of reflex-actions (Levine, 2007). Sherrington’s proposal for the brain architecture had its foundations on neuronal communication, strongly based on four principles: (a) neurons would transmit information “digitally” across long distances^1^ (i.e., action potentials) coding intensity as inter-pulse intervals—Adrian and Zotterman’s work (Adrian, 1926; Adrian and Zotterman, 1926); (b) the synapse would integrate the arriving “pulses” as postsynaptic “analog” potentials—decoding the frequency of the all-or-nothing “digital” input signals into signal intensity levels; (c) neurons could receive inputs from several synapses, coming from different sources, each either exciting or inhibiting postsynaptic potential formation, and; (d) depending on the level of excitation of the postsynaptic neuron, it could fire and propagate information. This whole rational scheme is named integrate and fire.

The foundational work of Sherrington—and his predecessors—was complemented by the important contribution, a couple of decades later, of the theoretical framework of Frank Rosenblatt and the McCulloch-Pitts neuron. In fact, while those previous conceptions were mostly based on inductive thinking proposed in order to develop a comprehensive theory of brain function, Rosenblatt’s implemented the McCulloch-Pitts “perceptron” as a means to test such a groundbreaking theory under a more deductive thought process made possible by mathematical models (McCulloch and Pitts, 1943; Rosenblatt, 1958). His attempt, named connectionism, was significantly different from the pure symbolic approach to “mimic” brain function without any concern for its biological substrate, defended by none other than Alan Turing (Daylight, 2015). On the contrary, the incorporation of neuroscientific ideas, such as Hebbian rules for learning and memory, largely contributed to the impact of such views in neuroscience in general, creating back-propagation models with a great impact on machine-learning systems (Lillicrap et al., 2020). Of particular importance here, it was also instrumental in laying down the later basis for ideas on how electrical stimulation could relay its effects, for example: (a) the higher the intensity of the current flowing through the stimulating electrodes, the higher the number of neurons recruited; (b) the higher the frequency of pulse stimulation, the higher the activation of each neuronal unit; (c) the pulse should obey the time-constraints known to the interpulse-interval associated to the “digital” propagation, i.e., the refractory period intrinsic to action potentials; (d) the target of the electrical stimulation should be chosen based on how its intrinsic functionality (localization v.s. function) could serve to alleviate symptoms, “remove” irritable areas (by either lesion or driving them into refractory periods), and potentiate endogenous feedback mechanisms to restore homeostasis (Beenhakker and Huguenard, 2009; Kalitzin et al., 2010).

Serrington’s work, although of undeniable genius and importance, was criticized and deemed incomplete especially due to its inability to properly address “the binding problem” and the temporal constrains that a cascade of integrator circuits would have on neuronal computation (Paz and Huguenard, 2015; Isbister et al., 2018). In short, not only the proposed conceptual framework struggled to explain how a “sensory-multimodal” object or process would be represented by the underlying neuronal network, but also that the neuronal computation of integrating neuronal frequency of discharge to an analog transmembrane potential in every synaptic relay would render complex sensory-motor integration (or decision making in voluntary movements) way too long a process to be useful in triggering behavior with an adaptive value. Therefore, adjustments should be made to the proposed framework regarding what kind of neuronal computational processes actually takes place within the brain. Nowadays we are on the verge of consolidating a new conceptual framework emerge: *time*. Time is the key element missing from the former early 20th-century debate.

### 2.2. Incorporating the coincidence detection architecture

The idea of a temporal structured organization, with neuronal circuitry designed to detect specific temporal discharge patterns, based on coincidence detector motifs rather than integrate and fire relays, has more recently (in the time frame of neuroscientific reasoning) been proposed as a conceptual framework alternative in neurosciences. Not that the current view completely erases previous conceptions, but rather that it complements ideas on brain physiology, in which an architecture based in space (i.e., anatomical-localizationism) on input areas (i.e., sensory systems) yields to a structure in time on more rostral substrates, allowing fast and reliable neuronal representation of the external world. Furthermore, at the output (i.e., rostral motor areas), the time-structured neuronal representations would gradually yield back to a framework based on a spatial organization, thus more akin to the integrate and fire paradigm. This central concept has received many different names/views and branched into many different ideas, all converging on the importance of time determining neuronal network organization and function: temporal-coding, phase-coding (Varela et al., 2001; Petersen and Buzsáki, 2020), phase-synchronization (Singer and Gray, 1995), time-synchronization (Grossberg and Schmajuk, 1989; Price and Gavornik, 2022), phase-coherence (Fries, 2005), amplitude-phase coupling (Aru et al., 2015), the neural syntax of brain rhythms (Buzsáki and Watson, 2012), spectral-signatures (Spadone et al., 2021), small-world network scheme (Liao et al., 2017), etc.

The very idea of a synchronous or coincident activity implies establishing the time-scale resolution deemed sufficiently small to be considered simultaneous, i.e., if it falls within the same time bin then it is synchronous. In fact, since multimodal information processing time-lags can vary throughout primary sensory pathways, due to the summation of sequential synaptic delays, a form of sustained activation state must exist in order to identify a “temporal pattern” of coincidental activity within increasingly larger time scales. That is the precise role fast brain oscillations are proposed to play within distributed local networks (i.e., neuronal assemblies) and the gradually decreasing frequency oscillations play on large-scale neuronal assembly integration (Varela et al., 2001). Consequently, the transient link that is established within local network assemblies (i.e., organized as small-world networks; Liao et al., 2017) and the dynamical large-scale transfer of information between such networks far apart in the brain have been referred to as a functional connectome (or connectogram)—in contrast with the quasi-static anatomical definition of a connectome (Contreras et al., 2015). One major corroboration of such perspective is the work done on selective attention and feature-binding neuronal visual processing, which has shown that phase-locking among neuronal groups (”temporal” coherence) plays an important role in efficacious communication between assemblies (Fries, 2005). In short, the same visual stimuli, applied during different attentional conditions, generate different temporal organizations amongst neuronal groups (not necessarily affecting “who” is being recruited—or “how much” that particular group is being recruited, but impacting “when” or in which sequence the neuronal groups are activated). The “binding-tag” could form transitory temporally coherent neuronal ensembles, creating a much more flexible and effective neuronal representation of a specific situation that could optimally trigger an appropriate sensory-motor response (Isbister et al., 2018).

Advances have also been made in proposing alternate mathematical models that are better suited to the idea of representation and communication by temporal organization of neuronal groups (i.e., binding-by-synchronization (BBS) hypothesis, communication-through-coherence, engram formation by temporal patterns). As an example, after observing precise firing sequences on task-triggered cortical activity, Abeles (Abeles, 1982; Abeles et al., 2004) coined the term “synfire-chain” to represent his idea of neuronal network organization based on “temporal patterns” of representation. Abeles “resurrected” previous models proposed by Griffith (1963) and Grossberg (Grossberg, 1969; Grossberg and Schmajuk, 1989) in which a version of Rosenblatt’s perceptron was reorganized into sequentially connected layers of pools of neurons, i.e., a “complete transmission lines”, each representing neurons that “synchronously” fired within a “hypothetical” time-bin. The “cortical songs”, represented by such a model, would have complex space-time patterns associated with “specific” network states (Louie and Wilson, 2001). The model received several modifications and suggestions along the years, such as no need for the time bins (or synaptic delays) to be constant—Bienenstock’s synfire braids (Bienenstock, 1995), and that synchronicity could be reinterpreted as a time-locked activity between neurons [not necessarily simultaneous firing (Izhikevich, 2006)]. These suggestions brought the proposed initial model much closer to current biological findings while also addressing criticisms against the model being able to provide an efficient, reliable, adaptive, and flexible representation of how the “real” world is represented by neuronal networks.

The aforementioned change in the conceptual framework of brain function and dysfunction has had a profound impact on conventional neuromodulation as a therapeutic and/or diagnostic tool. Patients with Schizophrenia have impaired performance on Gestalt-related tests (e.g., Mooney Face Test) that nicely correlates with large-scale neuronal synchronization deficits (Uhlhaas et al., 2006). On the other hand, forcing network coupling between two separate small-world networks, through electrotherapy-induced neuromodulation, has been shown to significantly improve impaired cognitive processes in patients (Reinhart and Nguyen, 2019; Reinhart, 2022). In short, in situations where physiological function and brain disorders just would not correlate with the changes in discharge frequency or metabolism of any one specific area, the overall temporal patterns between areas became a much more reliable diagnostic and therapeutic alternative. Furthermore, it has been shown that targeting a specific brain nucleus for ES, while using a pattern having the overall same frequency (i.e., 6 pulses within 100 ms), may activate different functional connectome pathways depending on the specific combination of IPI values used (Mourão et al., 2016). This particular result comes very close to demonstrating that the integrate and fire paradigm is not as well suited as the coincident detector one regarding higher-level neuronal processing.

All this theoretical framework substantially changed the way we looked at neurophysiology and, consequently, how the brain functions. There is an obvious two-way relation between how we perceive function and the strategy we design to fix things when they become dysfunctional; neuromodulation is no exception to that rule.

## 3. Novel conceptions of the time dimension in the design of neuromodulation approaches

A brief discussion on the use of neuromodulation for the diagnosis and treatment of patients with epilepsy is in order before we start suggesting paradigm shifts on the matter. Roughly, neuromodulation of targeted excitable nervous tissue has focused on varying parameters such as polarity, waveform, amplitude, frequency, electrode material, dipole distance among others in search of an optimal therapy (Medeiros and Moraes, 2014). The goal is often to cause disturbances on the underlying neural network in effect to its ongoing intrinsic state (McIntyre et al., 2004). Perhaps the most distinguished breakthrough in seizure control using neuromodulation happened during the 80s: vagus nerve stimulation (VNS) (Binnie, 2000). The VNS treatment was proposed as an effective method for treating patients with refractory epilepsy, found unfit for ablation surgery. The success of VNS prompted other more invasive neuromodulation approaches such as cortical stimulation and deep brain stimulation (DBS) (Kinoshita et al., 2004; Vonck et al., 2005). These alternatives were reserved for more extreme cases (i.e., patients with spasticity, severe psychiatric disorders, etc.). Tackling the problem through the development of new pharmacological agents had not significantly improved seizure control on pharmaco-resistant patients. Thus, the use of neuromodulation has regained the interest of both basic science epileptologists and clinicians. The new interest spurred a myriad of methods. Seizure suppressing neuromodulation has been applied, with different levels of success, to the anterior nucleus of thalamus (Mirski et al., 1997; Hamani et al., 2004), the subthalamic nuclei (Benabid et al., 2002; Chabardès et al., 2002), and even the epileptogenic focus itself (Vonck et al., 2002), including large clinical trials (SANTE) and with state-of-the art closed-loop systems (e.g., NeuroPace^®^ use in the treatment of refractory epilepsy, United States Food and Drug Administration) (Fisher et al., 2010; Morrell and RNS System in Epilepsy Study Group, 2011; Fridley et al., 2012).

It is not trivial to pinpoint the exact study or paper in which the time dimension for neuromodulation was first broadened into aspects beyond the integrate and fire framework and started affecting the design of the temporal structure of ES patterns. Although electrical stimulation has been used for rigorous scientific investigation of brain function for more than a century (Ferrier, 1887; Sherrington, 1906; Penfield and Boldrey, 1937)—see for review (Guenther, 2016), it was only after the birth of the modern era of neuromodulation in the ’60s, with DBS and Spinal Cord Stimulation (SCS) for the treatment of motor disorders and chronic pain (Buyten et al., 2013; Tiede et al., 2013; Chakravarthy et al., 2018), that its therapeutic usage started to share the same rigorous scientific foundations. Only then, the bidirectional knowledge transfer between basic neurosciences and neuromodulation truly deepened. Thus, it is natural that the first publications that more comprehensively reviewed the neurophysics of ES, such as the works of Ranck (1975) and Tehovnik (1996), were naturally committed to the integrate and fire framework. In these reviews, which are nonetheless bibliographic cornerstones to the field, the contribution of the temporal dimension for recruitment and control of behavior is described in terms of pulse frequency and duration, naturally conditioned to the target area. Even later work and theories aimed at understanding the mechanisms underlying therapeutic effects of DBS—such as depolarization blockade, synaptic inhibition, synaptic depression, and network modulation—were, to a large extent, linked to such framework as illustrated, for instance, to the prominent importance given to stimulation frequency (Breit et al., 2004; McIntyre et al., 2004; Theodore and Fisher, 2004; Vonck et al., 2004). In the same vein, much of contemporary work aimed at finding optimal parameters for DBS was focused on pulse morphology parameters, pulse frequency, and anatomical target (Kuncel et al., 2006; Cymerblit-Sabba et al., 2013), not on the temporal structure of stimuli.

Possibly, a major pioneering contribution toward better incorporating the time dimension into the design of neuromodulation strategies can be found in a series of *in silico* studies by Peter Tass and collaborators who, inspired by the phase resetting of circadian rhythms phenomena, set out to investigate analogous processes related to brain oscillations and stimulation (Tass, 1996, 2001a,b, 2002a,b). By using different mathematical-computational models of neural oscillators, that author and his group studied effects, mechanisms, and applications of pulsatile stimuli which are time-locked to ongoing synchronized oscillations in order to obtain phase resetting of the rhythm and thus desynchronization. Based on these findings, that same author later proposed the Coordinated Reset (CR) approach, which was a novel DBS variation to be applied in the treatment of neurological diseases such as Parkinson’s, motor disorders, and even epilepsy. Investigation of CR was first carried out *in silico* (Tass, 2003; Tass and Majtanik, 2006), and later in pre-clinical (Tass et al., 2012; Wang et al., 2016) and clinical settings (Adamchic et al., 2014) with considerable success.

In a parallel line of development, another important contribution stemmed from the efforts to better understand the mechanisms by which DBS is capable of suppressing motor symptoms of essential tremor. In 2004, Grill and colleagues put forward the informational lesion hypothesis to explain the therapeutic effects of high-frequency neurostimulation, stating that pathological activity would be masked by the input stimulus in a frequency-dependent fashion (Grill et al., 2004). In this *in silico* study, authors observed that the regularity of the firing of neurons increased (and thus the information content decreased) when increasing the stimulation frequency, which would corroborate two important DBS hallmarks: its efficacy in higher frequencies and its similarity to electrolytic lesions. This led the authors to postulate that application of irregular temporal patterns would be less efficacious in reducing essential tremor symptoms. This was, in fact, observed in a few following studies carried out *in silico* and also with human patients, by applying DBS in which IPI were drawn from Gaussian distributions with different coefficients of variation or in paired pulses (Birdno and Grill, 2008; Birdno et al., 2008, 2012). On the other hand, irregular temporal patterns—but of distinct features—of ES (uniform and unipeak distributions) were later demonstrated to improve the performance of Parkinsonian individuals in a finger-tapping task, while also suppressed aberrant electrophysiological spectral content in the beta band (Brocker et al., 2013). This set of results was of central importance in establishing the fact that temporal pattern is a determinant (a “new dimension”) in the efficacy of an ES method, even though effects may vary according to several other factors such as the disorder being treated, target area, and general parameters (Grill, 2018). In any case, Grill and colleagues have recently proposed new approaches (e.g., Temporally optimized patterned stimulation or TOPS^®^) in which parameters—including temporal—have been carefully engineered using, among others, machine learning tools (Brocker et al., 2017; Okun et al., 2022).

Considering the well-known aberrations of electrophysiological neural activity underlying epileptic phenomena in general and hyper-synchronism of seizures in particular (Avoli et al., 2002, 2004; Benini et al., 2003; Garcia et al., 2005; Nariai et al., 2011; Medeiros et al., 2014; Edakawa et al., 2016; Li et al., 2016; Abreu et al., 2018; Yu et al., 2018; Batista Tsukahara et al., 2022), epilepsy was also a natural application field for temporally complex ES. At the beginning of 2000’s, our group devised, patented, and tested—possibly the first therapeutically successful *in vivo* application of temporally structured ES—a novel temporally irregular pattern of ES later termed non-periodic stimulation (or NPS), in which the IPI were randomized in real-time, with the important advantage of being low frequency on average (mean of 4 pulses per second) (Cota et al., 2009, 2021; de Oliveira et al., 2019). Considering that therapeutic efficacy was obtained with pulse parameters compatible with other high-frequency methods, the low frequency directly implied low energy transfer from the stimulator to the neural tissue. This is highly advantageous for perspectives in engineering (greater autonomy of IPG batteries, less degradation of electrodes), medical practice (less interventions for battery substitution), and safety (lower risk of lesions and habituation) (Cota et al., 2016). In the first report on NPS anticonvulsant effects, seizures were induced in rats by the controlled intravenous infusion of pentylenetrazole (PTZ), a chemoconvulsant of broad action, while the occurrence of and latency to stereotypical convulsive behaviors were measured (Cota et al., 2009). Animals submitted to NPS applied to the right basolateral amygdala needed almost double the amount of PTZ to display generalized tonic-clonic seizures and displayed lower mortality levels when compared to fixed-frequency, burst, and quasi-uniformly distributed IPI patterns, as well as unstimulated controls. In an fMRI study using the same “ramp”-like infusion of PTZ, Mesquita and collaborators showed that the ipsilateral site of stimulation significantly increased activity during fixed frequency (also termed periodic) stimulation, while showing significant dampening of hyperactivity during NPS. These results confirmed the original 2009 data from Cota et al. (2009) and showed that the temporal dynamics of brain site activation during PTZ seizure onset was dependent on the temporal organization (inter-pulse-interval) of the same 4 pulses per second stimulation applied to the amygdala. In a way, NPS seemed to have a disruptive effect on the binding-by-synchronization dynamics of circuitry involved in the PTZ seizure model while the periodic stimulation facilitated and/or potentiated communication to the stimulated hemisphere.

The anticonvulsant effects of NPS were also demonstrated later in spontaneous seizures displayed by animals submitted to the temporal lobe epilepsy/chronic seizure experimental model of pilocarpine. After administering a bolus injection of the drug, animals develop status epilepticus which induces maladaptive plastic changes that will culminate in seizure susceptibility after a latent phase of 15 to 45 days (Turski et al., 1983; Clifford et al., 1987; Persinger et al., 1988). NPS-treated animals displayed fewer seizures, which were shorter and possibly less severe (Medeiros et al., 2014). Additional investigation also showed that the method is more efficacious if applied bilaterally and in an asynchronous fashion (Oliveira et al., 2018). While the anticonvulsant effects were confirmed by several mechanistic studies of our group (see next section) and other authors (see below), we also found preliminary evidence of beneficial effects in suppressing epileptogenesis and in the application towards pathological anxiety, which is mostly mediated by the amygdala (Cota et al., 2021). Finally, we recently demonstrated that neural function, including the sleep-wake cycle architecture, is preserved in animals undergoing NPS intervention (Réboli et al., 2022).

Among other groups that contributed to the investigation of non-standard temporal patterns of ES applied to the treatment of epilepsy, Wyckhuys and colleagues reported in 2010 the successful suppression of seizures in rats induced by kainate using ES with Poisson-distributed IPI at high frequency (130 Hz mean) (Wyckhuys et al., 2010). Quinkert and colleagues created non-standard temporal patterns of electrical stimulation with a mean frequency of 50 Hz by using a logistic equation and applied them to the hippocampus and medial thalamus of mice. They assessed behavioral and electrophysiologic biomarkers of arousal which may be related, by their turn, to disorders such as epilepsy or Parkinson’s disease. The authors found pattern-dependent behavioral alterations of increased arousal with concomitant increases in delta-range power of local field potentials. The effects were particularly strong with the non-linear patterns and depended on the applied substrate (Quinkert et al., 2010). Furthermore, following a sequence of studies aimed at developing closed-loop neurotechnology for the treatment of epilepsy, Nelson and colleagues also used ES with Poisson-distributed pulses to investigate the tolerability of different spatial and temporal regimes in multisite application to the cortex of rats with electrically-induced seizures. Authors found that synchronicity (temporal regime across areas) was more determinant in the suppression of seizures than the periodicity; fixed-frequency versus Poisson distributed IPI (Nelson et al., 2011). More recently, a temporal pattern similar to NPS was demonstrated to have, beyond anticonvulsant effects, anti-epileptogenic action in the amygdala kindling model in rats (Santos-Valencia et al., 2019).

All the studies discussed here show that the precise temporal structure of stimuli, beyond the fundamental concepts of frequency (or mean frequency) and pulse duration, is central in determining the effects, including therapeutic efficacy and collaterals, of a given neuromodulation approach. In our understanding, this should be considered a fact of past debate by now, even though several nuances remain to be elucidated. On the other hand, finding the precise common conceptual thread that binds all of them together into a unified scientific theory capable of explaining both brain function and the therapeutic efficacy of temporally structured ES, is challenging. In any case, we can benefit from some shared aspects of the neurological disorders and their neurostimulation methods discussed so far that, in our understanding, support the coincidence detection framework. In the next section of this manuscript, we will adopt this strategy, taking advantage of these ideas and also of the lessons learned from our own investigation of the mechanisms behind NPS, to put forward our attempt at a novel understanding of the mechanisms of temporally complex electrical stimulation. Naturally, this conceptual framework is largely based on coincidence-detection neuroscience introduced in earlier sections of this manuscript.

## 4. NPS meets the coincidence-detection framework: driving the infinite improbability of the brain’s functional connectogram

The choice of how to frame any problem, including brain function and dysfunction, has an obvious impact on how one attempts to solve it. Thus, the first step to designing efficacious and safe ES and understanding the mechanisms behind its therapeutic effects is to reinterpret the pathophysiology of brain disorders in a shared perspective of the coincidence-detection framework. In this sense, while normal levels of coupling within neural circuits and between areas of the brain are central for proper brain function (Womelsdorf et al., 2007), deviations from the natural setpoint may cause dysfunction (Uhlhaas and Singer, 2006). Some disorders, such as epilepsy, can be seen as abnormal coupling and indiscriminate propagation of information along neuronal pathways, without the apparent proper homeostatic modulation of inhibitory feedback mechanisms, thus compromising overall network stability (Moraes et al., 2000; Medeiros et al., 2014; Cota et al., 2021). Conversely, there are other cases, in which the core of the disorder seems to be related to the compromise of information transfer from one processing relay to the next (i.e., either by lesion or interference from other abnormally activated brain areas). An illustrative result, in this case, is the significant cognitive improvement found in age-related working memory deficits after “forcing” the coupling between brain regions using high-density transcranial alternating current stimulation (HD-tACS) ES. The dual-site HD-tACS was effective not only in reversing age-related cognitive deficits but also improved spatial task performance in adult human subjects (Zhang et al., 2022). In other words, by “grossly” stimulating two different brain areas using a non-invasive procedure, Reinhart and Nguyen were able to frequency tune theta wave synchronization along the frontoparietal cortex (Reinhart and Nguyen, 2019; Reinhart, 2022). If one considers gamma oscillation as the electrophysiological counterpart of local circuitry motifs (Hasenstaub et al., 2005; Vida et al., 2006; Bartos et al., 2007), then theta-gamma phase-amplitude codes would represent a long-range sender-receiver flow of information throughout the neural network; which was enhanced after the synchronized stimulation.

**FIGURE 1:**
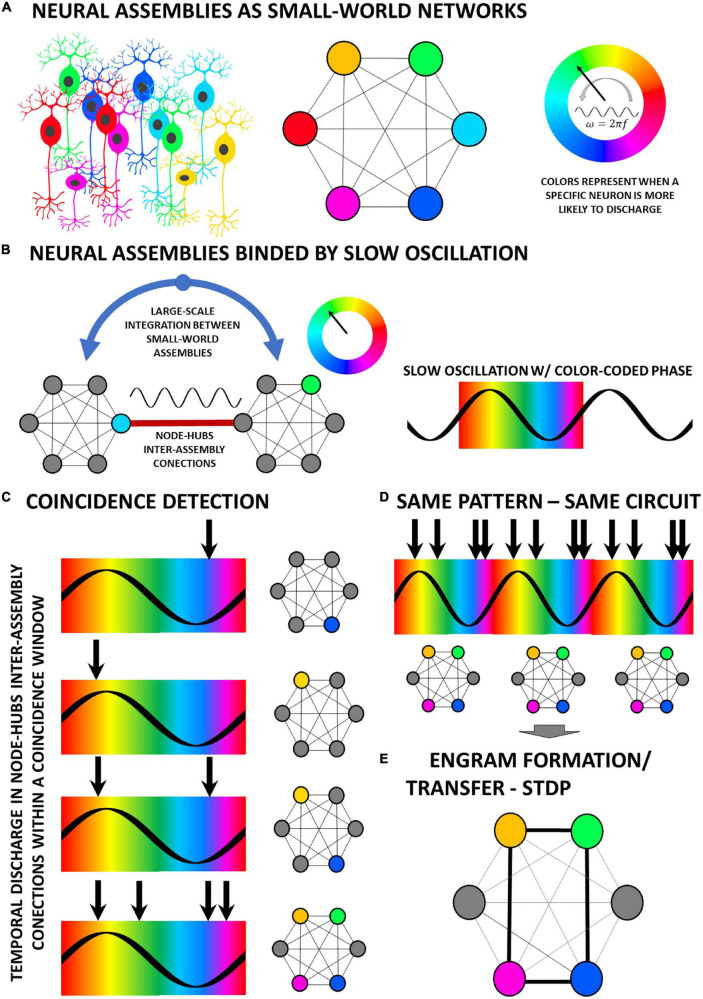
The time dimension in the processing of information across neural circuitry underlies the bidirectional interplay between temporal patterns of activity and the dynamic connectivity of neural circuits. **(A)** Neuronal motifs of multiple cells, codified by distinct colors, are organized as a highly interconnected network of nodes which are recruited in a time-basis locked to the oscillation phase, color-coded to match the motif which has a greater probability of recruitment. **(B)** Oscillations, which can be generated externally, synchronously entrain neural assemblies in a small-world network fashion for the transfer of information across long ranges (Varela et al., 2001). **(C)** By this coincidence detection scheme, the moment of occurrence of a given neuronal activity (e.g., firing of an action potential) in relation to the phase of the binding oscillation will determine which node will be activated in the afferent local network. **(D)** A specific temporal pattern coherently (to the phase of the slow oscillation) repeated over multiple cycles of reverberation will always recruit the same nodes in the local network. **(E)** Finally, the consistent spatiotemporal pattern of recruitment will induce, by means of mechanisms such as STDP, the creation of new engrams in the network.

The model of temporally structured information being transferred from one area to the next, as we saw in the BBS hypothesis or the communication-through-coherence, also requires a modulating oscillatory process phase-locking both regions. By creating “time-pockets” that favor activation of distinct and specific groups of neurons in the target network (i.e., in terms of propensity/probability to fire), in a selective fashion linked to the phase of the slow modulatory oscillation, distinct temporal patterns of discharge could be channeled through a particular node-hub from the origin—i.e., high degree participation coefficient nodes (Liao et al., 2017)—and thus would elicit an equally complex temporal and spatial activation pattern on the target network (Figure 1). Of particular importance here, this phase-locking scheme of neuronal communication between assemblies is only possible by looking through the coincidence-detection network framework. Following this logic, one could ask, what would happen, according to this scheme, if a random (or pseudo-random) distribution of discharge patterns is applied within a period of a coherent oscillation waveform between areas A and B? According to what was explained, a different and complex spatial and temporal pattern of activation on the target region would emerge every single time (Figure 1C). However, if a fixed pattern within an oscillation period were to be applied, then the same spatiotemporal pattern would be recruited over and over again in a reverberatory fashion (Figure 1D). Furthermore, this could result in its increasing control of the overall network activity due to Spike-Timing Dependent Plasticity—STDP (Feldman, 2012), eventually rendering the entrained circuit a self-sustained oscillator (Figure 1E).

**FIGURE 2:**
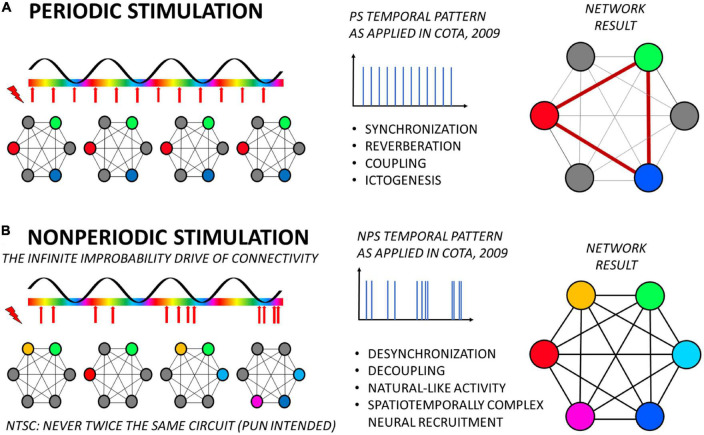
Temporally complex electrical stimulation applied to a hub-node (such as NPS target at the basolateral amygdala) benefits from the coincidence detection mechanism to deliver treatment by means of driving the functional connectivity of the brain with “infinite improbability”. **(A)** Periodic stimulation, with a fixed frequency (as seen in the center panel), always recruits the same nodes in the network and in the the same temporal order. This consistent spatiotemporal recruitment will thus induce a set of brain phenomena, such as synchronization (as observed by electrophysiology or fMRI), reverberation and creation of stereotypical electrographic signatures, increases in synaptic weights (coupling of nodes), and even ictogenesis in susceptible tissue. **(B)** Conversely, temporally complex stimulation, with continuously randomized IPI, induces an equally complex spatial (and temporal) recruitment of nodes in the network, never repeating the same circuit or the same order. By its turn, the lack of repetition will induce desynchronization, decrease of synaptic weights (decoupling), and thus robust anticonvulsant effects. If stimuli are organized as a natural-like activity, network response may be putatively tuned to homeostasis levels, rectifying synchronization to baseline, while being innocuous to neural function and acting in a demand-only fashion. It is important to highlight that the relationship between frequencies of stimuli and of the slow oscillation are not depicted to scale. Yet, the same effects on the consistency of the resulting recruitment of nodes in the network will probably remain unaltered given only that fixed frequencies can entrain the slow oscillation (e.g., by means of phase resetting) and temporally complex patterns can not. Naturally, investigation of these specific aspects is of paramount importance to better understand the validity of this framework.

The results we have been finding so far are evidence that this is precisely what happened when NPS produced anti-convulsant effects on animal models of epilepsy and periodic ES produced a proconvulsant effect (Cota et al., 2009; Figure 2). In other words, the therapeutic pattern resulted in a complex spatial and temporal activation of neuronal groups in the target and its afferent projections, thus preventing aberrantly synchronous recruitment of any specific neural circuitry into ictogenesis (Figure 2B). Conversely, using the same principles, simple low-frequency periodic activation resulted in the entrainment of the same network into pathological oscillation (Figure 2A). The imaging results using fMRI carried out by our group and discussed here (Mesquita et al., 2011) can be understood as first more direct evidence of that. Furthermore, functional characteristics of the stimulated neural substrate are naturally a major factor in these effects, as it happens with any other therapeutic application of ES, temporally complex or not. As mentioned, most of the findings are the result of the application of NPS to the basolateral amygdala, which is known to be a major node-hub connecting many territories in the forebrain, midbrain, and even hindbrain for the support of multiple neural functions (Antoniadis et al., 2009; Freese and Amaral, 2009), while also playing a major role in epileptic phenomena (Hirsch et al., 1997; Cota et al., 2016), directly or indirectly (de Oliveira et al., 2019). In the same vein, it is important to notice that more caudal structures in the primary-sensory relays may not be sensitive to such temporal organization of stimuli (Medeiros et al., 2012), while multiple site stimulation may contribute itself to uncoupling the coherence between large-scale information transfer (Oliveira et al., 2018).

Another important aspect that must be observed when considering the effects of NPS or other temporally complex approaches in the coincidence-detection perspective is that, in order to fit the idea of communication-through-coherence or BBS, the entire code generated by the ES pattern (i.e., the whole temporospatial representation within the network) must fit within a period of the oscillation in which communication is effective, whatever IPI distribution is chosen (Figures 1, 2). Not forgetting, as said before, e.g., the synfire chains, that layers upon layers of such time-coded representations could be combined into one single temporally structured sequence (i.e., “a cortical song”) representing a complex experience, object, qualia, or event. Nevertheless, the smallest unit of representation would still be organized within one oscillatory period of the “coupling” portion of the synchronization wave between two connected small-world- networks, thus allowing complex spatiotemporal representations to be channeled through node-hubs (i.e., optimizing neuronal processing and white matter taking up too much space). Importantly, in the end, this leads also to the understanding that frequency (in this case mean frequency) has also an important influence on the outcome of the pattern.

At this point, it is also important to recall that several of the proposed temporally complex ES protocols seem to have taken inspiration from the intuitive notion that temporal complex patterns of ES (random, non-linear, Poisson, and power-law distribution of IPI, etc.) mimicking physiological firing patterns of neurons would be able to resonate with neural circuitry and induce homeostatic physiological brain activity, suppressing aberrant sustained and/or high-intensity oscillations and promoting treatment. Such a concept has, in fact, inspired distinct studies non-related to therapeutic neurostimulation in the past. For instance, Gal and Marom used input-output joint statistics in order to assess the level of fidelity that a single synaptically-isolated neuron would respond to temporally complex stimuli (Gal and Marom, 2013). They have found that neuronal firing better reproduces input stimulation (optimal fidelity) when it is structured with natural-like, scale-free statistics, in which IPIs are distributed following a power-law of unitary exponent. These observations were later reproduced and extended to small neuronal networks *in vitro* (Scarsi et al., 2017).

Moreover, these aspects also served as a basis for speculating on the nature of many of our own results, particularly in the perspective of why just a specific form of temporal distribution is therapeutic (power law), while others are not (quasi-uniform) (Cota et al., 2021). A recent *in silico* study of our group using an amygdala-like network of Izhikevich neurons showed that power-law distributed stimulation more effectively recruited the local network into synchronized activity when compared to quasi-uniform (Oliveira et al., 2022). Particularly, in our proposed therapeutic temporal distribution, we were careful, as were other stimuli (Brocker et al., 2013), to limit the minimum IPIs to a value large enough to overcome integration time determined by membrane potential decay; i.e., not falling within a time-bin short enough for being considered coincident. Nevertheless, by choosing also an upper limit to the IPI distribution, Brocker and colleagues had much better results treating Parkinson’s; arguably making their distribution more alike a power-law distribution. On the other hand, it is also of paramount importance to recognize that different IPI distributions directly affect, as already mentioned, the mean frequency of stimuli or, when this parameter is controlled, its frequency content in distinct ranges of low versus high frequencies. Furthermore, other patterns very distinct from power-law or Poisson distributions (which are closely related to each other) have been shown to be efficacious in recruiting distributed local networks (Mourão et al., 2016). In fact, these data would corroborate the idea that normal base-line brain functioning would not favor one specific temporal arrangement over another; however, it would still depend on synchronizing such arrangements into timeframes established by oscillations (i.e., phase-coded processes) involved in the process, but not the pattern itself, of information transfer between nuclei. Finally, one has to consider the many differences across all these studies, such as the overall condition (homeostasis versus dysfunction) and the differences in experimental approaches (*in vivo* versus *in vitro* versus *in silico*). In the opinion of the authors, such a discussion is certainly related to a major knowledge gap in temporally complex ES. Hence, a thorough and careful investigation of the precise temporal structure of the ES must be carried out, with experiments specifically tailored to study the effects of all these aspects (IPI distribution, mean frequency, anatomical target) separately.

Looking at the problem from another angle, if the system has intrinsic transient “brain states” that favor abnormal coupling between brain areas, ES could be also used to probe how easily a signal from A would reach area B; thus, working quite effectively as a predictor of such brain-states. Medeiros et al. (2014) showed that a single pulse of ES applied every 2 s would be enough to synchronize spontaneous pre-ictal spikes long before any changes in parameters associated with passively recording EEG activity would be significantly different. Kalitzin et al. (2005) used probing ES to evaluate a measure (relative phase clustering index) of how much “synchronicity-prone” evoked activity existed between different regions of the brain as a form of predicting the emergence of seizures. Actually, probing ES as a surrogate marker of seizure onset was not only more effective than passively observing the electrographic activity of brain structures, but was also shown to be “plastically enhanced” if pre-conditioned to previous seizure episodes themselves (Medeiros et al., 2018)—i.e., the circuit learns to be a better biomarker if properly taught. These results not only show that the therapeutic, diagnostic, or predictive use of ES has very much shifted from the initial dogma that synchronicity is a consequence of excitability (Kudela et al., 2003), but rather that a myriad of new applications for ES arise if these two concepts (i.e., excitation/synchronicity) are untwined under the new proposed conceptual framework (Moraes et al., 2021). In fact, even the rationale for how some pharmacological targets are effective in treating epilepsy has been revisited under the coincidence-detector framework (Medeiros et al., 2020).

The intrinsic “brain states” mentioned at the beginning of the last paragraph, in some cases, could reflect structural abnormalities in the underlying neural network, with much more constant and permanent aftermath to the patients’ health (Uhlhaas and Singer, 2006). Uhlhass and collaborators showed that patients with schizophrenia not only did not perform well in the Mooney Face Test (i.e., Gestalt perception), but that the “communication-through-coherence” long-scale information transfer between brain structures was also compromised during the test. Several neuropsychiatric disorders (i.e., anxiety, depression, bipolar disorder, ADHD, sleep disorder/apnea, movement disorder/tremor), pain disorders (i.e., migraine, chronic pain, fibromyalgia, neuropathic pain), and other conditions could share a “gradual” commitment of neuronal network stability and inter-assembly communication deficits, within an intensity spectrum, with common physiopathological origins. It is not surprising that all these conditions are comorbid with what would be the extreme expression of abnormal coupling, network instability, and mass neuronal recruitment: epilepsy. Genetic animal models of epilepsy, possessing an innate propensity to seize, even if naive to having had a seizure, still display abnormal behavioral traits associated with the aforementioned comorbidities (Jobe et al., 1999; Jobe, 2003; Castro et al., 2017). And not only can brain-states compromise proper large-scale interactions, information transfer between small-world networks and modulate overall neural network processing, but specific temporal patterns of stimulation are required to induce brain-states [e.g., arousal (Quinkert et al., 2010)].

## 5. Discussion

Development and assessment of novel neurotechnologies in which the practice of experiments are tightly conditioned to the best of our knowledge of the underlying mechanisms is a major contributing factor for the fostering of impactful findings and disruptive technology (Sunderam et al., 2010; Cota and Moraes, 2022). We can envisage several perspectives for temporally patterned ES in general and also NPS in particular.

A major interest of our research has been epilepsy, due not only to its high morbidity and prevalence, but also because it can serve as an optimal platform for neuroscientific investigation and neurotechnolgical development. Although up to 70% of patients have seizure control with pharmacological treatment, a substantial number of individuals depend on surgical modalities for treatment (Sander, 1997; Brown, 2016; Johannessen Landmark et al., 2020). When it is not possible to identify the epileptogenic zone, or it is unresectable, the use of neuromodulation with electrical stimulation is an alternative for the reduction in the frequency and severity of seizures (George et al., 1994; Fisher et al., 2010; Heck et al., 2014; Velasco et al., 2022). Three modalities currently have devices commercially available for clinical use: VNS (George et al., 1994), DBS (Fisher et al., 2010), and RNS closed-loop responsive brain stimulation (Heck et al., 2014). Despite significant differences in the site and periodicity of stimulus administration, all use fixed frequency ES and show similar results with an approximate 50% reduction in seizure frequency in patients with focal refractory epilepsy (Ryvlin et al., 2021). They also use high-frequency fixed patterns with the main effect of putative direct target inhibition (functional injury) or activation interrupting pathological activity (jamming effect) of neural circuits (Carlson et al., 2010). On the other hand, robust evidence of therapeutic effects of using non-standard temporal patterns, from computational, animal, and human studies, has been accumulating over the years, as reviewed here. Together with the contribution of our own group, this support that NPS could also be an interesting alternative to increase the effectiveness of neuromodulation in the treatment of epileptic seizures, with a reduction in dysfunctions related to the effect of functional injury. Despite requiring hardware modifications, the adoption of this approach can be incorporated into clinical practice, as it does not increase the energy administered to the neural tissue, is reversible and can be applied to the same targets already used. As mentioned previously, NPS has been shown to be effective in dysfunctional tissue in animal models of the disease (de Oliveira et al., 2014), while also preserving neural function and the architecture of the sleep-wake cycle (Réboli et al., 2022). Both studies corroborate the translational potential of the method.

Despite the high morbidity of seizures, they represent only a small percentage of brain activity time. In this scenario, adoption of on-demand treatment measures is a logical path to pursue. Seizure prediction is pivotal to achieve this goal, but despite all the effort in trying to optimize such functionality using the recording of spontaneous brain activity, the time scale for anticipation is very small. Even with non-linear analyses, the prediction capacity usually does not exceed minutes, being debatable whether it is really a prediction or just early detection (Mormann et al., 2007; Andrzejak et al., 2009). The epileptic brain behaves as a complex system that, upon undergoing a critical transition, changes from a system resilient to hypersynchronism to a hypersynchronous and hyperexcitable system (Da Silva et al., 2003; Uhlhaas and Singer, 2006; Truccolo et al., 2011; Jiruska et al., 2013). More recently, algorithms that combine linear and non-linear analysis approaches have shown improvement in detection performance, although they still show large variability between individuals (Freestone et al., 2017; Karoly et al., 2017; Kuhlmann et al., 2018a,b). Thus, active probing of neural circuits, assessing the degree of resilience through stereotyped and predictable responses generated by external stimuli, can help detect critical transitions and favor better seizure detection (Moraes et al., 2021). With less effect of functional deficit associated with stimulation, NPS is also a promising technique in active probing, with the potential for more frequent circuit checks, using less energy. The lower functional deficit by suppression of local synaptic activity would cause fewer side effects in patients with non-ideally positioned electrodes. Targets where the functional deficit is unacceptable, could also be used.

If, in fact, temporally complex ES works by taking advantage of coincidence detection within the brain to recruit multiple microcircuits in the afferences of the node-hub target, a myriad of therapeutic possibilities ensues. The group is currently pursuing some of them by using the devised method NPS. Based on the rationale that the hyperfunction of the amygdala is directly related to pathological anxiety and/or chronic stress (Prager et al., 2014, 2016), we have been investigating therapeutic effects of NPS in animals submitted to stress model induced by chronic short-time confinement (Cota et al., 2021). We have also proposed its application to Parkinson’s disease (PD) and it is envisaged for the application in the suppression of aberrant activity displayed by animals submitted to a stroke model, this last in the realm of the EU-funded project MoRPHEUS. Understanding time-dependent events in the target neural circuit is critical to optimize parameters for activity disruption. Therefore, computational modeling and case analysis, fundamental in translating the method to greater applicability in clinical practice, is currently being carried out (Carvalho et al., 2021; Batista Tsukahara et al., 2022; Oliveira et al., 2022; Terra et al., 2022). Finally, spatiotemporally complex ES (NPS included) is a major plus if one considers the application of neuromodulation in a personalized or individualized fashion. Such approach is in line with the concept of electroceuticals in which neuromodulation therapy should be delivered in a manner that is finely tuned to the dysfunction, including individual patient particularities (Famm et al., 2013). In fact, several groups have been pioneering the application of stimulation in which the temporal pattern is optimized for therapeutic efficacy (Okun et al., 2022) and/or when the stimulation pattern resembles that of spontaneous neural activity (Cottone et al., 2018; Persichilli et al., 2022). Thus, besides anatomical target, frequency, pulse duration, phase, and amplitude, physicians will be able to choose different temporal patterns that may be better suited to different scenarios, encompassing the variability seen in distinct patients suffering from the same neurological disorder.

Overall, like in the Douglas Adams’ fiction—mentioned here simply to create a captivating analogy—in which zero-time space travel has been made possible due to an infinite improbability drive (a generator of randomness with infinite capacity), NPS applied to an important neural hub such as the amygdala creates ever changing temporal patterns of stimuli that would translate to ever changing recruitment of neural circuits or motifs and thus impair hypersynchronization; which, by its turn is characterized by excessive regularity. Differently from science fiction, the interchangeability between space and time in brain phenomena is a known and well-stablished scientific fact, with a powerful capability of explaining neural function and dysfunction, as we believe we made clear in this manuscript. We particularly envisage a future in which neurostimulation technology, enabled by closed-loop design and advanced-computing capability (e.g., neuromorphism; Chiappalone et al., 2022), will automatically choose among myriad parameters and also distinct temporal patterns (from fixed low frequency probing stimuli to high frequency random pulses) to deliver efficacious, efficient, and safe therapy. Further investigation of this promising strategy should be encouraged.

## Author contributions

VC and MM conceived the work and contributed the same to the writing of the manuscript. SC wrote smaller portions of the manuscript. All authors contributed to the article and approved the submitted version.

## Footnotes

1 Although the electric-field propagation within a conducting volume does allow for synaptic communication if the distances are sufficiently small (i.e., electrotonic conduction, electrical synapses, ephaptic transmission etc.), in order to overcome the constraints imposed by physics on electric field decay, a biological solution based on voltage-gated channels, working roughly as “repeater stations”, allow for signal transmission over meter-long axonal processes.
